# Association of psychological resilience and social support with subjective coercion experience in hospitalized psychiatric inpatients: a cross-sectional study

**DOI:** 10.3389/fpubh.2026.1833344

**Published:** 2026-06-25

**Authors:** Xinyu Shi, Jianying Yu, Xiaohong Gong, Leiyu Yue, Biru Luo, Yu Zhuo

**Affiliations:** 1 Department of Nursing, West China Second University Hospital, Sichuan University/West China School of Nursing, Sichuan University, Chengdu, China; 2 Key Laboratory of Birth Defects and Related Diseases of Women and Children (Sichuan University), Ministry of Education, Chengdu, China; 3 Mental Health Center of West China Hospital, Sichuan University, Chengdu, China

**Keywords:** coercion, mental disorder, psychological nursing, root cause analysis, social support

## Abstract

**Background:**

Coercive medical measures, such as physical restraint, compulsory medication, and seclusion, are common in psychiatric hospitalization and may negatively impact patients' autonomy and psychological wellbeing, triggering subjective coercion experience. This study aimed to describe the current status of coercion experience among psychiatric inpatients and identify associated influencing factors, with a focus on psychological resilience and social support.

**Methods:**

This cross-sectional study recruited 161 psychiatric inpatients from child/adolescent and adult wards of a tertiary hospital in Chengdu, China. Subjective coercion, psychological resilience and social support were assessed using the Chinese CES, CD-RISC, and SSRS. Multiple linear regression analysis was used to identify factors related to coercion experience.

**Results:**

Univariate analysis showed that patients with lower educational attainment reported significantly higher CES scores (*P* < 0.05). Both psychological resilience and social support were negatively correlated with coercion experience (r = −0.355, *P* < 0.001; r = −0.178, *P* < 0.05). In multiple linear regression, psychological resilience remained a significant negative predictor of subjective coercion experience (*P* < 0.001).

**Conclusion:**

Subjective coercion experience among psychiatric inpatients was negatively associated with psychological resilience. These findings suggest that psychological resilience may help buffer the impact of coercive interventions. Further research is needed to inform strategies for reducing negative coercion experiences in psychiatric inpatient settings.

## 1 Introduction

Mental disorders are complex diseases characterized by cognitive, emotional, and behavioral impairments, resulting from a combination of genetic, environmental, and social factors (1, 2). During hospitalization, psychiatric patients are commonly subjected to coercive measures (3), including compulsory medication, seclusion and mechanical restraint (4). In a study of 1764 adult psychiatric inpatients in a Swiss open-ward hospital, 16.6% experienced at least one seclusion or restraint (5). Among children and adolescents, a systematic review showed a median prevalence of 17.5% for any coercive measure in inpatient child and adolescent mental health services (6). These measures can harm both the physical and mental health of patients. Studies have shown that mechanical restraint can lead to cardiac deterioration and even cardiac arrest and death (7), and coercive measures are significantly associated with a decline in mental health status at discharge (8). Patients often describe these experiences as terrifying, humiliating, and re-traumatic, especially among those with a history of abuse, which may undermine their trust in mental health services (9–11). When patients perceive that their personal freedom has been violated during the application of coercive measures, they are prone to negative stress, perceived rejection and negative emotions such as fear, anxiety, and shame (12, 13). These subjective feelings arising from undergoing intervention against one's will is known as coercion experience (14). Studies have shown that patients' coercion experiences can have short-term effects including increased symptoms of post-traumatic stress disorder, decreased satisfaction, and poorer clinical condition (15, 16). In the long term, various forms of coercion may also be associated with lower functional improvement and increased risk of readmission (17). Therefore, reducing coercive measures and alleviating unavoidable coercion experience may help reduce trauma, stigma, and service disengagement, and may indirectly reduce re rehospitalization rates (16, 17).

The use of coercive measures in psychiatric care poses significant ethical challenges for nurses. At the core of this dilemma lies the balance between respecting patient autonomy and fulfilling professional care responsibilities, which includes both beneficence (promoting the patient's wellbeing) and non-maleficence (avoiding harm) (18, 19). Promoting the patient's best interests is the primary justification for coercion, yet respecting patient autonomy remains a fundamental challenge whenever coercive measures are applied (18). Recent ethical studies have also developed the concept of relational autonomy, which defines autonomy not simply as individual self-determination but as the ability to shape and direct one's life within relationships with others (20, 21). From this perspective, measures aimed at enhancing patients' long-term autonomy may be ethically distinct from unjustified paternalism.

The subjective coercion experience is now widely recognized as involving acute psychological distress, and potentially leading to long-term trauma and challenges to recovery (9, 16). Some studies have shown that patients' response to coercive interventions are influenced not only by the measure itself, but also by a variety of situational factors. These include the quality of the clinician-patient relationship, the patient's participation in treatment decisions, and the overall ward atmosphere (22, 23). Further research has confirmed that if patients feel respected by healthcare professionals and have ample opportunities to participate, their coercion experience and subsequent psychological trauma can be effectively alleviated (9). Based on these findings, recovery-oriented and trauma-informed care models have gradually developed. These models focus on protecting patients' autonomy, building a positive clinician-patient relationship, and helping patients accumulate personal and social support resources to enhance their adaptability and coping abilities (24, 25). While situational factors such as the attitude of healthcare professionals and the overall ward atmosphere are crucial, these external conditions are not entirely within the patients' control. In contrast, internal psychological resources such as psychological resilience and subjectively perceived social support are equally important, helping patients proactively regulate their state and alleviate the psychological discomfort caused by coercion.

To clarify the influence of various individual factors on coercion experiences and the underlying psychological mechanisms, this study drew on the stress process model developed by Pearlin et al. (26). This model provides a basic analytical framework for explaining the pathways through which external stress induces psychological distress, highlighting the mediating role of social support and coping resources. Its core logic is that individuals can proactively mobilize their internal and external resources to buffer the negative impact of coercion on their mental health (27). Over the past forty years, the stress process model has been validated in different populations and various stressors, stably and reasonably explaining the process by which stress factors induce psychological distress in different situations. Related research has also continuously improved the applicability and theoretical framework of this model (28, 29).

When applying the stress process model to research on coercion experience in psychiatry, the experience can be understood as a dynamic process formed by the interaction of three elements: external stressors, mediating resources, and psychological outcomes. Coercive interventions are external stressors, and patients often perceive them as threat to their autonomy and dignity (16). This subjective perception further impacts core dimensions of self-concept, including self-esteem and a sense of control (26), leading to negative emotions such as fear and anxiety. Critically, the core idea of this model is that individual and societal resources can interrupt this negative development process. Psychological resilience is an individual's positive adaptability in the face of adversity (30), which can alleviate the negative emotions brought about by coercion experiences through stabilizing self-concept and improving coping skills. Social support can provide emotional comfort, help individuals restructure their cognition, and view stressful situations from a more positive perspective, thereby maintaining their mental health (31, 32).

Relevant evidence indicates that social support and positive coping strategies can reduce patients' psychological stress and support recovery within psychiatric settings (27, 31). However, empirical studies specifically exploring how psychological resilience and social support alleviate the subjective coercion experience in hospitalized psychiatric patients remain relatively limited. Furthermore, most relevant research findings come from Western countries, where mental health service systems, family involvement patterns, and public cultural attitudes toward patient autonomy may differ from those in China. Therefore, the applicability of the findings in different cultural contexts needs further examination. In this context, this study aimed to (a) compare coercion experience across demographic and clinical characteristics in a Chinese psychiatric inpatient sample, and (b) examine the extent to which psychological resilience, social support, and sociodemographic factors are associated with coercion experience.

## 2 Materials and methods

### 2.1 Study participants

This cross-sectional study included psychiatric inpatients through convenience sampling from December 2023 to March 2024 at the mental health center of a tertiary hospital in Chengdu, Sichuan Province, China. Participants were recruited from three closed inpatient wards: one child and adolescent ward and two adult wards. As the mental health center encompasses both a child and adolescent ward and adult wards within a single facility, the sample spans a broad age range. Patients from psychogeriatric, dementia, and neuropsychiatric specialty wards were excluded.

Participants eligible for this study were (a) diagnosed as mental and behavioral disorders according to the Chinese Classification of Mental Disorders or Diagnostic Criteria ICD-10; (b) those who had experienced at least one coercive medical measure during the current hospitalization, as documented in the nursing records and clinical notes; (c) able to understand and complete the questionnaire with assistance if needed; and (d) those with informed consent and admitted in this study voluntarily. Coercive measures are defined in detail in Section 2.2.

Patients were excluded if they had (a) serious comorbid physical disorders, dyslexia, or documented organic brain disease/intellectual disability; or (b) cognitive impairment or communication difficulties that would interfere with study participation. The research team assessed each patient's comprehension ability through clinical judgment during daily interactions, focusing on their understanding of the study, the informed consent process, and sample questionnaire items.

This study used a sample size calculation method for estimating the overall mean, setting it as a two-sided test with α at 0.05. It was known through literature review that the expected standard deviation was 30 and the allowable error was 5 (33). The sample size N=139 cases was calculated by using PASS 15.0 software. Considering a 15% loss to follow-up rate, a total of 161 samples were finally required for this study.

### 2.2 Operational definition of coercive medical measures

The distinction between formal coercive measures and ward-level restrictions adopted in this study draws on the conceptualization of coercive measures as existing on a continuum of severity, ranging from persuasion and interpersonal leverage through inducements and threats to formal compulsory treatment (34, 35). At the most restrictive end of this continuum, formal coercive measures (mechanical restraint, seclusion, and compulsory medication) involve direct bodily intrusion or confinement. These interventions correspond to the definition of formal coercion as interventions that are formally regulated, documented, and that restrict freedom of movement in psychiatric inpatient care (18). While ward-level management restrictions, including limitations on mobile phone use and on leaving the ward, are relatively mild forms of coercion, they still limit patients' personal autonomy and raise corresponding ethical issues (35). This study does not make a simple binary division of coercive measures, but rather uses this operational definition to clearly analyze the different effects of formal coercive measures and ward-level restrictions on patients' subjective coercion experience. This approach is especially relevant in Chinese psychiatric inpatient settings, where ward-level policies such as mobile phone restrictions are common and represent a meaningful dimension of patients' daily experience of restricted autonomy.

### 2.3 Instruments

#### 2.3.1 Demographic and clinical characteristics

Sociodemographic variables included gender, age and degree of education. The clinical information included the medical diagnosis of the current disease (filled in by researchers based on the patient's medical record), whether the admission was voluntary, and what type of coercive measures were received after hospitalization.

#### 2.3.2 Coercion Experience Scale, CES

The Chinese version of the Coercion Experience Scale (CES) measures patients' coercion experiences across six dimensions: rights limitation (perceived loss of autonomy and freedom), passivity (felt lack of control or forced compliance), negative environment (unpleasant or hostile surroundings), adverse physical effects (physical discomfort or harm), negative reactions (emotional distress such as anger or humiliation), and fear (anxiety or threat to safety). The dimension content was summarized based on the explicit meaning of each item. In clinical practice, the Chinese version of CES has proved its good reliability and validity, with a Cronbach's α coefficient of 0.965 and a split-half co-efficient reliability of 0.858 (13).

In this study, we evaluated the internal consistency reliability of the Chinese version of CES across its six dimensions. For these four dimensions, Cronbach's α coefficients were as follows: Rights Limitation (Cronbach's α = 0.901), Passivity (Cronbach's α = 0.885), Negative Environment (Cronbach's α = 0.783), and Adverse Physical Effects (Cronbach's α = 0.784), all indicating good internal consistency. For the two-item dimensions (Negative Reactions and Fear), we calculated both Cronbach's alpha and the Spearman-Brown coefficient, as the Spearman-Brown coefficient is a more appropriate reliability coefficient to be reported for a two-item scale (36). The Cronbach's α coefficients were 0.672 and 0.500, and the Spearman-Brown coefficients were 0.675 and 0.500. These values suggested acceptable reliability for Negative Reactions and Fear when dimensions were assessed with only two items. Overall, the CES demonstrated satisfactory reliability in this sample.

#### 2.3.3 Connor-Davidson Resilience Scale, CD-RISC

The Connor-Davidson Resilience Scale consists of 25 items, including three dimensions: resilience, self-improvement, and optimism. The revised full scale has good reliability and validity, and its Cronbach's α coefficient is 0.91 (30). In the current sample, after testing, the Cronbach's α for the CD-RISC was 0.956.

#### 2.3.4 Social Support Rating Scale, SSRS

The Social Support Rating Scale was developed by Chinese scholar Xiao Shuiyuan (37). This scale is designed briefly and contains only ten items, covering three dimensions: objective support, subjective support, and the utilization of support. After examination, this scale has high reliability and validity, and the test-retest reliability r = 0.92 (37). In this sample, the Cronbach's α coefficient of the social support rating scale was 0.785.

### 2.4 Ethical considerations

This study was approved by the Ethics Review Committee of West China Hospital of Sichuan University (Approval Number: 2023-2281). All personally identifiable patient information was anonymized. Data were securely encrypted and stored, with access restricted to members of the research team. Before the formal survey began, the researchers explained in detail to the patients and their legal guardians the purpose of the study, the use of the data, and the potential risks, ensuring that all participants were fully informed and voluntarily involved in the survey. Researchers adjusted their communication methods and explanations according to the patients' comprehension abilities to ensure clear and understandable information delivery. During data collection, if a patient exhibited emotional distress or clear resistance, the questionnaire survey was immediately terminated, and appropriate emotional support were provided. Psychological support intervention was offered when indicated. Both guardians and participants retained the right to withdraw from the study at any time without any effect on the latter's ongoing medical care.

### 2.5 Statistical analysis

**Figure 1:**
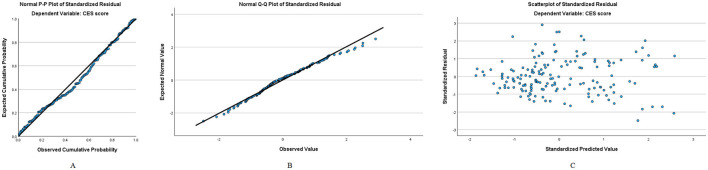
(**A)** Normal P-P plot of standardized residual; **(B)** Normal Q-Q plot of standardized residual; **(C)** Scatterplot of standardized residual.

(1) Statistical description: The countable data were expressed as frequency or percentages; The measurable data conforming to normal distribution were expressed as mean ± standard deviation, and the measurable data not conforming to normal distribution were expressed as median (interquartile range). (2) Statistical analysis: Univariate analyses were performed using the Mann-Whitney U test (for two independent samples), Kruskal-Wallis H test (for two independent samples), and Pearson correlation analysis (for continuous variables). Variables with *P* < 0.05 in any of these univariate analyses were entered into a linear regression model. To ensure the validity of the regression model, residual normality, homoscedasticity, and multicollinearity were assessed. The residuals were approximately normally distributed, as indicated by the P-P and Q-Q plots (Figures 1A, B), as well as non-significant Kolmogorov-Smirnov (*P* = 0.061) and Shapiro-Wilk (*P* = 0.130) tests. Homoscedasticity was confirmed by a scatterplot of standardized residuals against predicted values (Figure 1C), which showed random dispersion without any funnel-shaped patterns. Multicollinearity diagnostics was assessed with variance inflation factors (VIF) ranging from 1.094 to 1.507 and tolerance values exceeding 0.6.

All statistical analysis was completed with SPSS 29.0 software. This study was conducted and reported in accordance with the STROBE guidelines (38).

## 3 Results

### 3.1 Univariate analysis of the influencing factors of coercion experience

A total of 161 inpatients with mental disorders were investigated in the Mental Health Center of West China Hospital, Sichuan University, of whom 37 were male (22.98%) and 124 were female (77.02%). The youngest was 12 years old and the oldest was 59 years old. The univariate analysis of the remaining data is detailed in Table 1.

**Table 1:** Univariate analysis of the levels of coercion experience of in hospitalized psychiatric patients.

Variables	Groups	Sample size, *n*	Coercion experience	Z/H	*P*	Effect size (r/η^2^)
Gender	
	Male	37	62.00 (56.00, 77.00)	1.539 (Z)	0.124	0.121
	Female	124	71.00 (57.00, 98.75)			
Age, year	
	≤ 17	103	71.50 (57.00, 94.50)	1.080 (H)	0.583	0.006
	18–45	52	65.50 (56.00, 93.00)			
	>45	6	59.00 (50.00, 91.50)			
Degree of education	
	Bachelor's degree or above	39	66.00 (56.00, 100.00)	9.936 (H)	**0.019**	0.044
	High school or technical secondary school education	45	62.00 (53.00, 74.50)			
	Middle school	68	74.00 (58.00, 97.75)			
	Primary school	9	80.00 (70.50, 106.50)			
Diagnosis	
	Depressive disorder	73	71.00 (58.00, 97.50)	3.651 (H)	0.455	0.002
	Schizophrenia	16	62.00 (55.25, 77.00)			
	Bipolar affective disorder	36	63.00 (48.25, 99.75)			
	Childhood emotional disorders	22	70.50 (59.75, 96.25)			
	Anxiety disorder	14	69.50 (61.75, 88.50)			
Voluntary admission	
	Yes	117	64.00 (54.00, 82.00)	−3.919 (Z)	**< 0.001**	0.309
	No	44	84.50 (62.50, 114.50)			
**Type of coercive measures**	
	Ward-level restriction	117	64.00 (54.00, 79.00)	21.956 (H)	**< 0.001**	0.132
	Formal coercive measure	44	91.00 (67.25, 114.50)			

Bold values indicate statistical significance at *P* < 0.05.

### 3.2 Correlation analysis of psychological resilience, social support and coercion experience

The total score of the CD-RISC was (43.35 ± 20.67), and the average score of each item was (1.73 ± 0.83). The total score of the SSRS was from 9 to 48, with a median score of 23.00 (19.00, 26.00). The results of Pearson correlation analysis showed that the correlation value between psychological resilience and patients' coercion experience was −0.355 (*P* < 0.001); the correlation between social support and patients' stress experience was −0.178 (*P* < 0.05), as shown in Table 2.

**Table 2:** Correlation analysis of psychological resilience, social support and coercion experience.

Variables	Total CES score	Rights limitation	Passivity	Negative environment	Physical adverse effects	Negative reactions	Fear	Psychological resilience	Social support
Total CES score	1								
Rights limitation	0.857^***^	1							
Passivity	0.953^***^	0.748^***^	1						
Negative environment	0.847^***^	0.569^***^	0.787^***^	1					
Physical adverse effects	0.796^***^	0.552^***^	0.767^***^	0.685^***^	1				
Negative reactions	0.738^***^	0.566^***^	0.650^***^	0.653^***^	0.541^***^	1			
Fear	0.660^***^	0.439^***^	0.613^***^	0.541^***^	0.548^***^	0.493^***^	1		
Psychological resilience	−0.335^***^	−0.178^*^	−0.305^***^	−0.394^***^	−0.276^***^	−0.281^***^	−0.391^***^	1	
Social support	−0.178^*^	−0.053	−0.136	−0.239^**^	−0.241^**^	−0.167^*^	−0.267^***^	0.520^***^	1

CES, coercion experience scale; ^***^P < 0.001, ^**^P < 0.01, ^*^P < 0.05.

### 3.3 Multiple linear regression analysis of the influencing factors of coercion experience

Taking the total score of coercion experience as the dependent variable and the categorical data with statistical significance in univariate analysis and correlation analysis as the independent variables, a multiple linear regression analysis of the influencing factors of coercion experience in hospitalized psychiatric inpatients was conducted. The details are shown in Table 3 and Table 4.

**Table 3:** Assignment table of independent variables.

Variables	Assignment description
Degree of education	1 = Bachelor's degree or above; 2 = High school or technical secondary school education; 3 = Middle school; 4 = Primary school
Voluntary admission	1 = Yes; 2 = no
Type of coercive measures	1= Ward-level restriction; 2 = Formal coercive measure
Psychological resilience	Enter with the original value
Social support	Enter with the original value

**Table 4:** Multiple linear regression analysis of the influencing factors of coercion experience.

Independent variables	*B*	Standard deviation	*Beta*	*t*	*P*	**95% CI. of** ***B***	
						Lower limit	Upper limit
(Constant)	57.430	13.294		4.320	< 0.001	31.170	83.690
Degree of education	−0.373	2.200	−0.013	−0.170	0.866	−4.718	3.972
Voluntary admission	11.307	4.313	0.194	2.622	**0.010**	2.787	19.827
Type of coercive measures	16.667	4.229	0.286	3.941	**< 0.001**	8.312	25.021
Psychological resilience	−0.303	0.104	−0.240	−2.917	**0.004**	−0.508	−0.098
Social support	−0.109	0.285	−0.032	−0.382	0.703	−0.671	0.454

R = 0.506, R^2^ = 0.256, Adjusted R^2^ = 0.232, F = 10.676, *P* < 0.001. Bold values indicate statistical significance at *P* < 0.05.

## 4 Discussion

This study indicated that psychological resilience, admission status, and the type of coercive measures significantly influenced the subjective coercion experience among hospitalized psychiatric patients. Consistent with prior research, psychological resilience was negatively associated with CES scores (39). Importantly, involuntary admission is a key predictor of patients' subjective coercion experience, a finding consistent with previous research indicating that loss of autonomy during hospitalization affects patients' psychological state (16, 22). Furthermore, patients receiving formal coercive measures scored significantly higher on the CES compared to those receiving ward-level restrictions. This result corroborates evidence that invasive interventions intensify perceived coercion and provoke negative emotional responses such as fear and humiliation (9, 15). Social support was correlated with coercion experience in the univariate analysis but did not remain significant in the regression model. This finding is consistent with a previous study (40) but contradicts other studies (27, 41). The difference may stem from the mediating effects of psychological resilience and social support on the coercion experience, which could be further explored in the future using structural equation modeling. In addition, lower education levels were linked to higher coercion experience scores, which aligns with Gu's findings (42). This association may stem from a limited understanding of therapeutic procedures and fewer personal resources for processing coercive events.

Overall, these findings are consistent with the stress process model (26) and the Buffering Hypothesis (27). Within these two theoretical frameworks, the coercion experience can be viewed as a dynamic process in which coercive measures, as external stressors, gradually contribute to distress through long-term chronic stress (such as role conflicts or loss of autonomy) and a weakened self-concept (such as decreased self-esteem). Psychological resilience may mitigate the stress transmission in the early stages by strengthening coping abilities (43), while social support may operate in the later stages, helping patients to reinterpret the stressful situations and restore their sense of self-worth and control (44).

These findings also have important ethical implications for clinical practice. There is a contradiction in the use of coercive intervention in psychiatric hospitalization: on the one hand, medical staff have the responsibility to ensure the patients' safety (the duty of care), and on the other hand, they need to respect patient autonomy. This contradiction is difficult to completely resolve. After all, in acute situations, a certain degree of coercion cannot be avoided (18). Nurses are the main professionals of coercive measures such as mechanical restraints, seclusion, and compulsory medication, and are in a key position in the whole process. Systematic reviews have pointed out that nurses often have anxiety, guilt and other emotions in their hearts when implementing such coercive measures, and at the same time fall into moral conflict, but they also know that these measures are needed to maintain the normal safety order of the ward (45, 46). In the face of such a real dilemma, it is necessary to explore the modifiable factors to reduce the subjective harm caused by coercive interventions.

Our finding that psychological resilience was negatively associated with subjective coercion experience may be relevant to clinical practice. This suggests that in the process of implementing coercive interventions, adopting resilience-oriented nursing strategies, such as encouraging patients to participate, safeguarding their human dignity, and maintaining open and transparent communication, may offer a way to reduce the ethical pressure of inevitable coercion. Relevant study also confirms that psychiatric nurses will take the initiative to increase the participation of patients during coercive procedures to maintain their autonomy and dignity (47), which is consistent with the finding of this study. It is also in line with the relational autonomy framework, which defines autonomy not as total freedom from external interference, but as the ability of individuals to shape their own lives within supportive relationships (21). Furthermore, studies have shown that nurses' personal safety may affect the probability of their coercive intervention (45), and different professional roles, stigma levels and working environments will cause different attitudes of medical staff toward coercive measures (48). Whether the interventions of improving resilience and encouraging participation can effectively reduce subjective coercion experience still needs to be verified by subsequent longitudinal research. Medical institutions continue to carry out ethical education, trauma-informed nursing and recovery-oriented training, which can also provide necessary support for nursing staff to deal with such inherent contradictions.

This study provides ideas for public mental health practice and relevant policy formulation in China, by identifying psychological resilience, admission status, and type of coercive measures as factors significantly associated with subjective coercion experience. Limited to the cross-sectional design, these findings cannot determine the causal relationships between variables, but the conclusions can generate hypotheses for follow-up intervention research. From the individual and family levels, digital resilience training, mindfulness intervention and other relevant programs to improve psychological resilience (49, 50), combined with structured family mental health education (51), may help patients and their support networks better cope with coercive situations. At the institutional and system level, reducing invasive intervention that clinical conditions allow (52), strengthening the training of emotional guidance ability and trauma-informed communication skills of medical staff (53), and improving the collaboration mechanism between hospitals and communities (54), may alleviate coercion-related distress and strengthen continuity of recovery-oriented care. From the current situation of China's medical system, further optimizing the integrated service model of hospital-community is a direction that can be promoted in the future. Currently, China's mental health system relies primarily on hospitals as service providers, with relatively limited rehabilitation and follow-up services at the community level (55). This model may increase the risk of readmission to some extent and trigger crisis-driven mandatory interventions. It is suggested that establishing community mental health centers that integrate case management, peer support, and family psychological education may help provide continuous care and reduce the incidence of acute hospitalizations (56). Given the exploratory and cross-sectional nature of this study, these recommendations should be viewed as hypotheses rather than definitive conclusions. Further research could employ prospective and interventional study designs to empirically verify whether interventions that combine individual psychological resilience development with supportive environments can effectively reduce the distress caused by coercion, while also providing feasible empirical evidence for clinical practice and policy formulation.

## 5 Limitations

This study still has some limitations. First, the cross-sectional study design made it impossible to clearly define the causal relationship between psychological resilience, admission status, type of coercive measures, and subjective coercion experience. Second, the sample in this study only included patients from a specific tertiary hospital, and the convenience sampling method was used to recruit participants, which not only reduces the applicability of the research conclusions but may also introduce selection bias. The sample was predominantly female (77.0%) and biased toward adolescent and young adult patients (64.0% aged ≤ 17 years). This profile likely reflects the timing of data collection in late winter and early spring, when hospitalization rates for seasonal affective disorders rise among female patients (57), together with growing mental health service utilization among Chinese adolescents (58). Therefore, these findings may not fully represent older male patients or populations in different healthcare systems. Third, the lack of age-stratified subgroup analysis is also a limitation, mainly because the adult sample size (*n* ≈ 58) is insufficient to effectively analyse a multivariate model containing five predictors, and this study was not designed to detect age-related effect modification, therefore no subgroup analysis was performed.

Finally, several aspects of the measurement and modeling approach warrant caution. Although a two-tier operational classification was used to distinguish formal coercive measures (mechanical restraint, seclusion, compulsory medication) from ward-level restrictions (mobile phone restriction and restriction on leaving the ward), heterogeneity within the formal category in particular may have obscured measure-specific effects, given the substantial differences in intrusiveness among these interventions. The relatively modest explained variance (adjusted R^2^ = 0.232) further suggests that unmeasured variables, such as patient-staff relationship quality, ward atmosphere, duration of hospitalization, number of previous admissions, and prior trauma history, may also contribute meaningfully to subjective coercion experience.

Future research should employ a multi-center longitudinal design, expanding the sample size and stratifying by age to examine differences in the associations of coercion experience with factors across different developmental stages. In addition to self-reported data, observational or clinician-assessed measures should be included to evaluate coercion experience more objectively. Furthermore, structural equation modeling could also be employed to examine the mediating and moderating pathways proposed by the stress process model, which may supplement the mechanisms not revealed by multivariate analysis.

## 6 Conclusion

This study employed a cross-sectional design to analyze the influencing factors related to the subjective coercion experience among hospitalized psychiatric patients. The results showed that patients with lower levels of psychological resilience, those admitted involuntarily, and those subjected to formal coercive measures (mechanical restraint, seclusion, and compulsory medication) had higher coercion experience scores. Univariate analysis indicated a negative correlation between social support levels and patients' coercion experience; however, this association was not significant in the multivariate model, suggesting that social support's impact on coercion experience is not direct and is likely influenced by other variables. Given the observational design, these associations should not be interpreted as causal. The modest explained variance further suggests that additional unmeasured variables likely contributed to how patients experienced coercive interventions. Future longitudinal and multi-center studies are needed to clarify the temporal relationships among these factors and to evaluate whether resilience-oriented and trauma-informed interventions can reduce the subjective distress associated with unavoidable coercive measures in psychiatric inpatient care.

## Data Availability

The raw data supporting the conclusions of this article will be made available by the authors, without undue reservation.
